# Challenges of modelling TDP-43 pathology in mice

**DOI:** 10.1007/s00335-025-10131-1

**Published:** 2025-04-29

**Authors:** José Miguel Brito Armas, Lucas Taoro-González, Elizabeth M. C. Fisher, Abraham Acevedo-Arozena

**Affiliations:** 1 Unidad de Investigación Hospital Universitario de Canarias, Instituto de Investigación Sanitaria de Canarias, CIBERNED and ITB-ULL, Tenerife, Spain; 2 Department of Neuromuscular Diseases and Queen Square Motor Neuron Disease Centre, UCL Queen Square Institute of Neurology, London, UK

## Abstract

TDP-43 is a normally nuclear RNA binding protein that under pathological conditions may be excluded from the nucleus and deposited in the cytoplasm in the form of insoluble polyubiquitinated and polyphosphorylated inclusions. This nuclear exclusion coupled with cytoplasmic accumulation is called TDP-43 pathology and contributes to a range of disorders collectively known as TDP-43 proteinopathies. These include the great majority of amyotrophic lateral sclerosis (ALS) cases, all limbic-predominant age-related TDP-43 encephalopathy (LATE), as well as up to 50% of frontotemporal lobar degeneration (FTLD) and Alzheimer’s disease (AD) cases. Thus, TDP-43 pathology is a common feature underlying a wide range of neurodegenerative conditions. However, modelling it has proven to be challenging, particularly generating models with concomitant TDP-43 loss of nuclear function and cytoplasmic inclusions. Here, focussing exclusively on mice, we discuss TDP-43 genetic models in terms of the presence of TDP-43 pathology, and we consider other models with TDP-43 pathology due to mutations in disparate genes. We also consider manipulations aimed at producing TDP-43 pathology, and we look at potential strategies to develop new, much needed models to address the many outstanding questions regarding how and why TDP-43 protein leaves the nucleus and accumulates in the cytoplasm, causing downstream dysfunction and devastating disease.

## Introduction

TDP-43 is an RNA binding protein belonging to the hnRNP family encoded by the *TARDBP* gene. TDP-43’s central role in disease pathogenesis comes from its identification as one of the main components of inclusions seen in brains from ALS and FTLD patients (Neumann et al. 2006). Now, primary TDP-43 pathology is the main feature defining > 98% ALS cases; the remaining cases are chiefly familial SOD1-ALS and FUS-ALS (Nolan et al. 2020). It also defines up to 50% of FTLD (excluding mutant *MAPT* (TAU) patients (Irwin et al. 2015) and is apparent in diseases arising from mutations in progranulin (*GRN*) and *C9Orf72*, as well as all LATE cases (Nelson et al. 2019), and other diseases such as multiple system proteinopathy, Perry syndrome and inclusion body myositis (IBM) (de Boer et al. 2020). Secondary TDP-43 pathology also occurs in other disorders, such as up to 50% of AD, where its appearance correlates with cognitive decline (Chang et al. 2023). This secondary pathology also occurs in cases of Huntington’s (Nguyen et al. 2025) and Parkinson’s disease, as well as in chronic traumatic encephalopathy. The primary and secondary TDP-43 pathologies are collectively known as TDP-43 proteinopathies (de Boer et al. 2020).

At the cellular level, the two main features characterising TDP-43 proteinopathies are its nuclear exclusion (or the appearance of nuclear aggregates/condensates), coupled with its cytoplasmic mislocalization and the formation of cytoplasmic inclusions. It is not clear how these two features arise, or how they result in TDP-43 proteinopathies, i.e. how they cause a pathological cascade that can elicit cell death. However, at the molecular level, a direct consequence of TDP-43 nuclear loss of function is pleotropic mRNA processing abnormalities, which produce generalised splicing dysfunction, including the appearance of cryptic exons (CE) such as within *Stathmin 2* (*STMN2*) or *UNC13A* genes (Melamed et al. 2019; Brown et al. 2022; Ma et al. 2022; Mehta et al. 2023).

### Why do we need more models?

As is the case with other major neurodegenerative diseases, TDP-43 proteinopathies are complex disorders for which there are no perfect models displaying all the major pathological features characterizing each disease. Taking ALS as a paradigmatic example, the defining feature of the disease is the dysfunction and loss of both upper (in motor cortex and brainstem) and lower motor neurons (in the spinal cord). Although all patients share these critical disease-defining features, there is a lot of clinicopathological heterogeneity within patients in terms of disease onset (bulbar versus spinal), disease duration (fast versus slow progressors), or genetics (sporadic versus familial, with more than 30 causative genes identified so far) (Fisher et al. 2023). It is therefore likely that different disease trajectories leading to motor neuron degeneration coexist, exemplified by the large number of genes mutated in ALS and their disparate functions. Thus, it is not possible to model all this complexity in single animal (or in cellular) models.

### Different folds, different pathologies, different models?

Modelling the appearance of TDP-43 in cytoplasmic inclusions coupled with the loss of the nuclear protein, has proven to be challenging in animals and in cellular models, with myriads of models producing one aspect of TDP-43 pathology, but not the other; for example, some overexpression mutant models might lead to inclusion formation, but not loss of function, whereas downregulation models would lead to loss of function without accompanied cytoplasmic inclusions. Thus, in part due to the lack of appropriate models, it has proven difficult to disentangle the differential contributions of both disease features in TDP-43 pathogenesis. In the last few years, TDP-43 loss of function alone, leading to pleiotropic mRNA processing defects including the appearance of CE, have emerged as critical mediators of toxicity (Mehta et al. 2023). Now, essential new data has started to emerge from different TDP-43 proteinopathies showing that cytoplasmic TDP-43 inclusions can adopt different protein folds in different human diseases. For example, in FTLD, TDP-43 pathology has been subdivided at least into four categories (FTLD-TDP-A to -D), depending on where the phosphoTDP-43 (pTDP-43) inclusions are present within the cortex and/or affected neurites (Neumann et al. 2021). Recent data shows that different TDP-43 folds, resolved by cryogenic electron microscopy (Cryo-EM), underlie TDP-43 inclusions present in different FTLD-TDP subtypes, suggesting a link between specific TDP-43 aggregates and their clinical manifestations (Arseni et al. 2022, 2024), and showing unanticipated heterogeneity in this disorder. It remains an open question as to whether the different folds are *required* to develop each FTLD-TDP subtype, leading onto the possibility that we might need models with specific TDP-43 folds to study the different FTLD-TDP subtypes and possibly other TDP-43 proteinopathies. Indeed, developing new models with specific TDP-43 folds is likely to play a key role in future research aiming at understanding their possible causality.

## Existing TDP-43 mouse models

Developing suitable in vivo models that will allow us access to in vivo biology—including the intricate interactions between various cellular populations that are believed to be crucial in the various TDP-43 proteinopathies—is essential for a better understanding of the disease processes at work in patients. However, from the plethora of TDP-43 mouse models available, only very few develop *bona fide* TDP-43 inclusions—with currently unknown folds—and TDP-43 overexpression is usually required to develop this pathology.

Here, we provide a comprehensive list of current TDP-43 mouse models available, curated by the presence of the different aspects of TDP-43 pathology (Table 1). As is clear from this list, despite an extensive set of models, very few develop the four critical aspects of TDP-43 pathology: (1) cytoplasmic inclusions, (2) nuclear loss of function leading to (3) splicing defects, all underlying specific neurodegeneration (4).

**Table 1:**
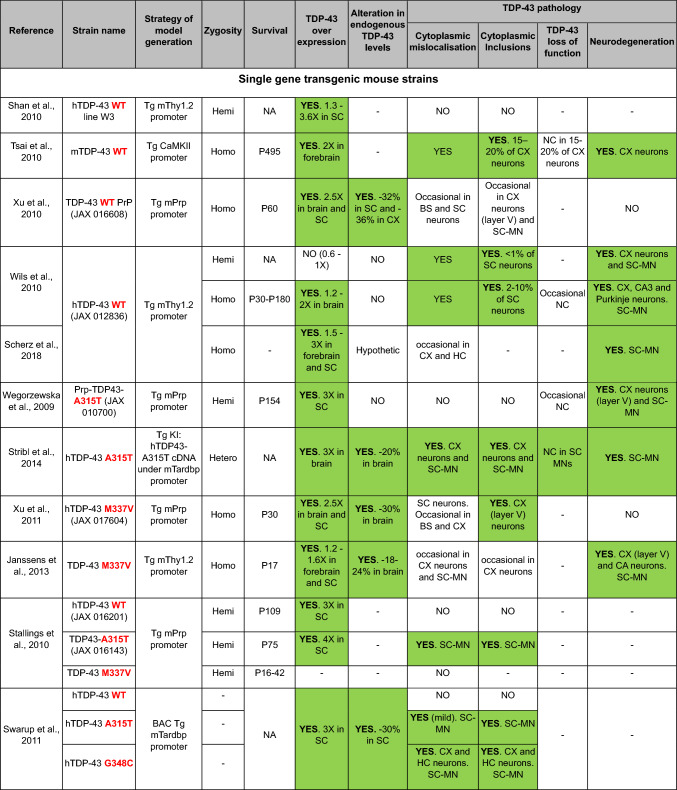

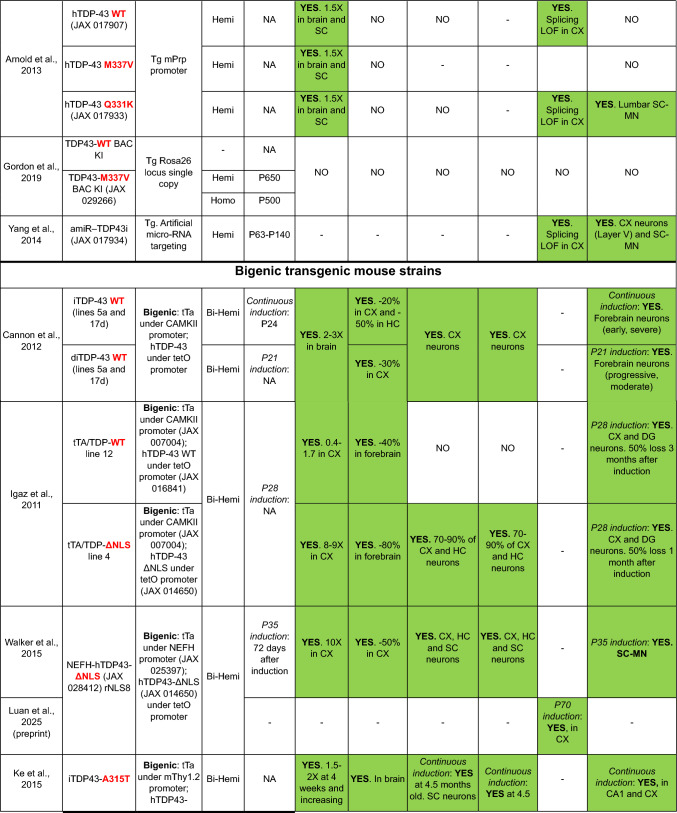

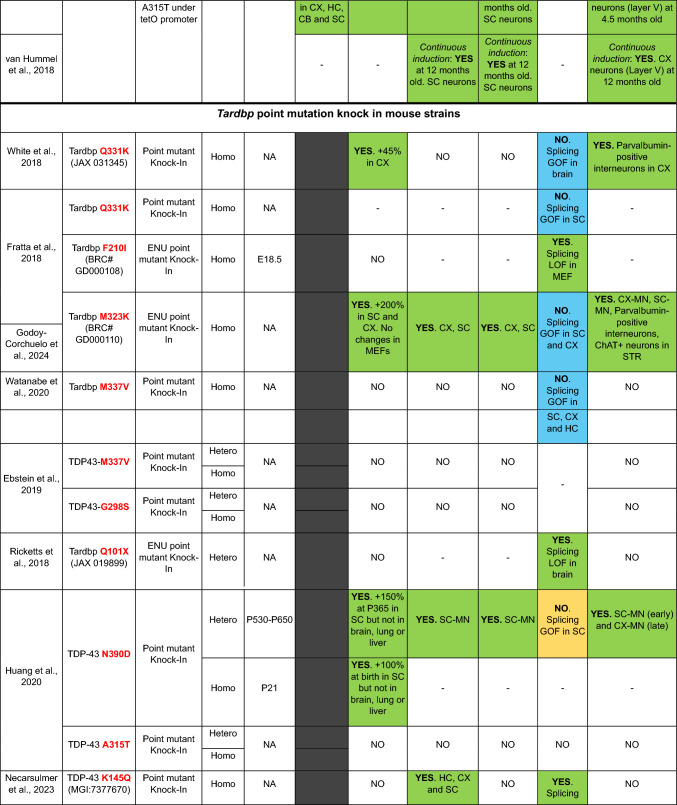

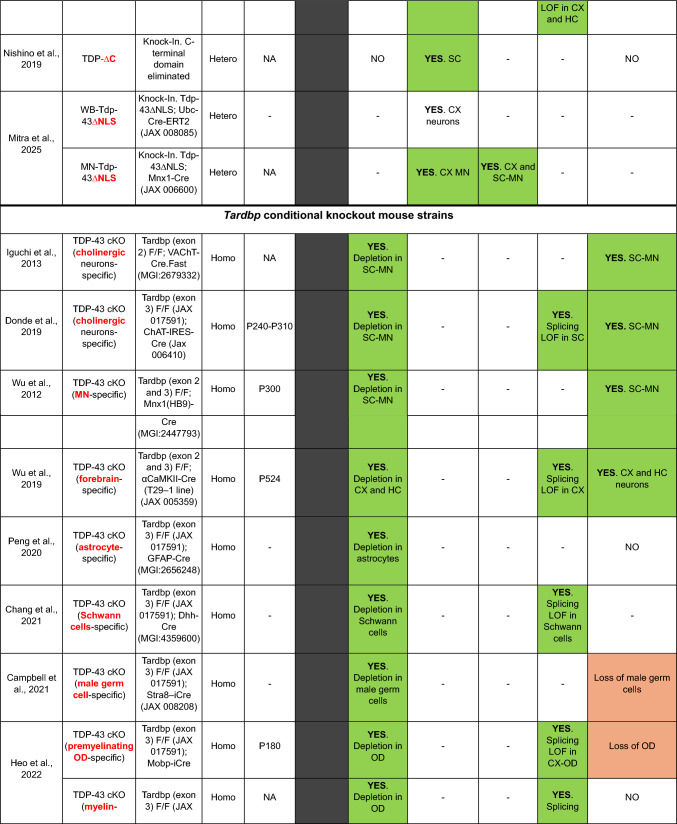

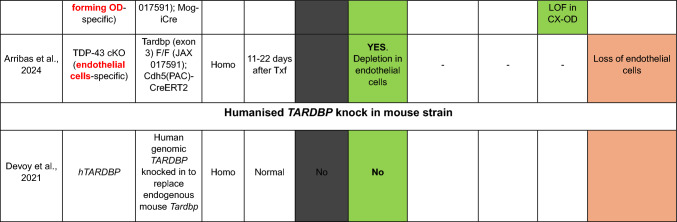
TDP-43 mouse models

In the table, models are grouped into five sections according to the generation strategy: single transgenics, bigenic inducible transgenics, point mutation knock-ins (KIs), conditional knockouts (KOs) and genomically humanized knockins. Within each section, models are chronologically ordered based on the introduced mutations (in transgenic and KI mice) or the targeted region or cell type (in conditional KO mice), highlighted in red and bold in both cases

BAC, bacterial artificial chromosome; Bi-Hemi, hemizygous for 2 transgenes; BS, brainstem, CA1, *cornu Ammonis* subfield 1; CA3, *cornu Ammonis* subfield 3; CB, cerebellum; ChAT, choline acetyltransferase; cKO, conditional knock-out; CX, brain cortex; DG, dentate gyrus; ENU, N-ethyl-N-nitrosourea; GOF, gain of function; HC, hippocampus; Hemi, hemizygous; Hetero, heterozygous; Homo, homozygous; KI, knock-in; LOF, loss of function; MN, motor neuron; MEF, mouse embryonic fibroblast; NA, non-affected; NC, nuclear clearance; OE, overexpression; OD, oligodendrocytes; SC, spinal cord; STR, striatum; Tg, transgenic; tTa, tetracycline transactivator transgene; tetO, tetracycline operator; Txf, tamoxifen; MEFs, mouse embryonic fibroblasts; –, not tested

### Transgenic TDP-43 models

Transgenic models are typically made by the random integration of a transgene (generally derived from a plasmid or bacterial artificial chromosome (BAC)) into the mouse genome. Thus, the two endogenous mouse alleles encoding TDP-43 (*Tardbp*) are intact. Furthermore, because transgenes tend to concatenate as they integrate into the host genome, these transgenes are present in multiple copies and therefore usually result in overexpression of the protein from the ectopic locus into which they have integrated (and which they may mutate) (Goodwin et al. 2019).

From the wide variety of available transgenic models, including wildtype (WT) gene overexpressors and those with human gene mutations such as A315T, M337V, Q331K or G348C (Table 1), there are a few useful lessons that arise. Firstly, in general, human TDP-43 overexpression, regardless of whether WT or mutant, can be toxic to neurons when expressed over a certain threshold, making it a challenge to separate the toxicity due to increasing TDP-43 expression levels, from the possible pathogenicity of mutations (Wegorzewska et al. 2009; Wils et al. 2010; Tsai et al. 2010; Arnold et al. 2013). Secondly, toxicity is not necessarily accompanied by the appearance of TDP-43 pathology, as is the case with many TDP-43 transgenic models that express WT or mutant protein and develop neurodegeneration but without TDP-43 cytoplasmic inclusions or nuclear loss (Wegorzewska et al. 2009; Wils et al. 2010; Igaz et al. 2011; Arnold et al. 2013; Janssens et al. 2013). Thirdly, prominent TDP-43 cytoplasmic inclusions might also occur without accompanying neurodegeneration, as in G348C BAC transgenics (Swarup et al. 2011). Thus, at least in some mouse models, toxicity might be linked to TDP-43 dysregulation and uncoupled from TDP-43 pathology. Lastly, TDP-43 overexpression leads to TDP-43 splicing gain of function, and not to the TDP-43 loss of function that defines end and possibly mid-stage disease in ALS patients (Irwin et al. 2024). Therefore, loss of function toxicity is generally not modelled by overexpressing transgenic models even under conditions of downregulation of the mouse endogenous alleles through autoregulation (Carmen-Orozco et al. 2024). Overall, the majority of overexpressing transgenics are appropriate models to study the effects of TDP-43 gain of function due to increasing expression levels and its toxic consequences, but it is not yet clear what role this might play in disease pathogenesis.

Nevertheless, probably the best current examples in terms of modelling all aspects of TDP-43 pathology are mice carrying a doxycycline (Dox)-suppressible transgene under a tetracycline-responsive promoter element (tetO) harbouring a human TDP-43 with a mutated nuclear localization signal (NLS) leading to its cytoplasmic localization (tetO ΔNLS)—which can be switched on and off depending on the availability of doxycycline in the diet by crossing this mouse to lines carrying a tetracycline transactivator (tTA) transgene under the control of different promoters, such as the CamK2a promotor that directs expression to forebrain regions (Igaz et al. 2011), or the human neurofilament heavy chain (*NEFH*) promoter, that gives expression in neurons in brain and spinal cord in rNLS8 mice (Igaz et al. 2011; Walker et al. 2015), the latter one including a diagram of the breeding strategy). In these NLS bigenic mice, carrying the two different transgenes, Dox treatment represses mutant hTDP-43 NLS transgene expression in neurons under the control of the specific promoter. After removing Dox from the diet, the mutant hTDP-43 NLS is turned on, leading in the rNLS8 mice to extensive TDP-43 pathology underlying motor neuron degeneration accompanied by a downregulation of mouse endogenous TDP-43 that leads to some TDP-43 loss of splicing function (Luan et al. 2025). Interestingly, non-cell autonomous effects have been also described in non-neuronal cells in rNLS8 mice, including microglia (Spiller et al. 2018) and oligodendrocytes (San Gil et al. 2024). Another model using the same inducible tetO NLS strain but crossed to mice expressing a rtTA transgene under the human skeletal actin promoter (HSA) also shows the expression of muscle specific CE along with TDP-43 cytoplasmic aggregates upon doxycycline treatment. Moreover, these mice showed that seed competent TDP-43 can persist in muscle in mice, correlating with the seeding capacity detected in muscle lysates from IBM but not ALS patients (Lynch et al. 2024).

Thus, these inducible NLS mice display all four critical aspects of TDP-43 pathology: nuclear loss of function leading to aberrant splicing coupled with cytoplasmic inclusions all leading to neurodegeneration—making them excellent models to study the consequences and the possible reversibility of all aspects of TDP-43 pathology. However, these mice, as with all models, have some limitations: they are constrained by the expression of the accompanied tTA transgene in bigenic mice. In rNLS8 mice, hTDP-43 is only expressed in neurons because it is driven by the *NEFH* promoter, and therefore it is not possible to ascertain whether there are cell autonomous defects in other critical cellular populations involved in ALS pathogenesis, such as glia, oligodendrocytes or skeletal muscle. Moreover, both human and mouse TDP-43 proteins are present (albeit at reduced levels for the mouse protein), which may influence TDP-43 functions and/or potential folding variations if there are species-specific differences in protein folding that have yet to be examined in vivo.

Transgenic models have also been used to assess the possible involvement of cellular pathways thought to be involved in disease pathogenesis by crossing them with reporter lines. hTDP-43 transgenic mice have been crossed with reporter mice expressing inflammation/oxidative stress (*NRF2*-*Hmox1* reporter) and senescence/DNA damage (*p53*–*p21* reporter) transgenes, showing an early activation of the in vivo reporters in parvalbumin-positive Purkinje and basket cells of the cerebellum (Ferro et al. 2024). These and other in vivo reporter models are likely to be useful to assess how TDP-43 pathology in any mouse model might affect particular cellular pathways.

### Viral TDP-43 mouse models

An alternative to transgenic mice are viral models, in which TDP-43 (*TARDBP*) is transduced directly into the brain using viral vectors, usually adenoviruses. These models have the advantage that using local injections into different brain areas leads to TDP-43 inclusions, allowing for studies of the spread of pathology from a single focus. Using these models, mutant TDP-43 has been shown to propagate in both directions through corticospinal circuits and to transfer into oligodendrocytes (Tsuboguchi et al. 2023). However, as these models rely on ectopic expression, they also have similar limitations as transgenic models, leading to TDP-43 overexpression in the context of the mouse endogenous alleles. Nevertheless, they are useful models to dissect the cellular consequences of TDP-43 dysfunction in specific cellular populations, and to assess TDP-43 propagation.

### TDP-43 Knock in (KI) mouse models

To try to circumvent the problems associated with overexpression, as TDP-43 levels are critical for its functions in health and disease, several KI models that mutate the mouse endogenous *Tardbp* gene have been developed by us and others (De Giorgio et al. 2019). In general, *Tardbp* KI models develop mildly progressive phenotypes due the endogenous levels of expression, and might be particularly appropriate to study early disease stages, with neurodegeneration only appearing in a minority of models, and usually only in homozygosis, whereas the majority of human *TARDBP* mutations are dominant—therefore, homozygous mutations might not directly model human disease (De Giorgio et al. 2019). An advantage of mutant KI models, as they affect the mouse endogenous alleles, is that they uniquely allow for testing the possible effects on TDP-43 loss of function of specific mutations.

In terms of TDP-43 pathology, only one strain, carrying the N390D mutation, has been reported to develop TDP-43 inclusions accompanied by neurodegeneration in heterozygosis (Huang et al. 2020). Why this particular strain develops neurodegeneration as well as TDP-43 pathology remains an open question, particularly as the model has not been further reported in the literature. Another KI model with neurodegeneration carries the M323K mutation in homozygosis, developing progressive upper and lower motor neuron degeneration coupled with TDP-43 cytoplasmic enrichment, but no TDP-43 positive inclusions, together with anatomical brain abnormalities (Godoy-Corchuelo et al. 2024). We have used this strain to show that at least some C-terminal *Tardbp* mutations can lead to TDP-43 splicing gain of function, resulting in the expression of skiptic exons even in cellular populations where TDP-43 expression levels are not affected (Fratta et al. 2018). Although it does not develop neurodegeneration, the K145Q mutation in homozygosis, mimicking the effects of TDP-43 acetylation, has a partial loss of RNA binding and the accumulation of insoluble TDP-43 in the brain, underlying progressive behavioural phenotypes but without any motor function abnormalities (Necarsulmer et al. 2023). The Q331K mutation in homozygosis also develops progressive behavioural phenotypes underlined by neuroanatomical abnormalities, and has been used to show that specific cortical parvalbumin interneurons are susceptible to neurodegeneration upon mutant TDP-43 expression, despite not presenting with motor neuron degeneration (White et al. 2018; Lin et al. 2021). Finally, a new conditional model carrying the deletion of a 16 amino acid stretch (∆82–98) within the N-terminus of the protein including the region containing the NLS, has been recently reported, showing that motor neuron specific expression of the deletion leads to TDP-43 cytoplasmic aggregates in motor neurons and skeletal muscle, accompanied by a decrease in neuronal markers and motor abnormalities – although it is not yet clear if the cytoplasmic mislocalization could lead to loss of nuclear TDP-43 function and the expression of CE (Mitra et al. 2025). This is a promising new model with potential to contribute to the understanding of how the different TDP-43 pathology factors interact, whilst avoiding some of the undesirable side effects from overexpression or downregulation.

Overall, all these data from different KI models show that, at least in the mouse, not all *Tardbp* mutations might be equal, adding an extra layer of complexity to the challenges of disease modelling. Moreover, although the majority of mutations are on a C57BL/6 genetic background, other backgrounds are also used, such as the C57BL/6J-DBA mixed background used in the M323K KI model, that might also contribute to phenotypic variation due to the presence of genetic modifiers.

### *Tardbp* loss of function models

Conditional *Tardbp* Knock Out (KO) mice are primarily used to model TDP-43 loss of function, because constitutive KO are not viable from very early during development (Sephton et al. 2010; Chiang et al. 2010), and heterozygous KO animals develop mild motor abnormalities without neurodegeneration due to autoregulation (Kraemer et al. 2010; Ricketts et al. 2014). Conditional KO are useful for dissecting the contribution of TDP-43 loss of function to toxicity in different cell types, having been used to show critical functions for TDP-43 in motor neurons (Donde et al. 2019), oligodendrocytes (Heo et al. 2022), skeletal muscle (Vogler et al. 2018) or germ cells (Campbell et al. 2021) amongst other cellular populations. In terms of TDP-43 pathology, conditional KO do not model TDP-43 cytoplasmic accumulation, but clearly model TDP-43 loss of function. They have been extremely useful for understanding the in vivo consequences of TDP-43 loss of function, in fact, they were used for the first identification of CE, leading to the subsequent identification of generally non-conserved CE in patients (Ling et al. 2015). In particular, the conditional deletion of *Tardbp* in motor neurons using the ChAT promoter shows that motor neurons are susceptible to TDP-43 loss of function, leading to progressive motor deficits underlined by motor neuron loss and the appearance of CE, but also showing that mice without TDP-43 in cholinergic motor neurons can survive for a surprisingly long time, to almost a year of age (Donde et al. 2019), perhaps highlighting the possible roles of TDP-43 toxicity in other cell types that might modulate the cell autonomous TDP-43 effects in motor neurons.

Interestingly, conditional KO mice have also been used to show critical functions for TDP-43 cytoplasmic aggregation during normal tissue homeostasis. The conditional deletion of *Tardbp* in satellite cells showed that TDP-43 cytoplasmic localization is required for correct skeletal muscle regeneration underlined by the formation of TDP-43 positive cytoplasmic myogranules (Vogler et al. 2018). Thus, at least during skeletal muscle regeneration, some cytoplasmic TDP-43 localization is required, showing that not all cytoplasmic aggregation of TDP-43 is pathological.

RNAi against *Tardbp* has also been used in transgenic mice to develop a model expressing generalized reduced amounts of endogenous *Tardbp*, leading to neurological as well as systemic effects. In the spinal cord, RNAi expression seems to primarily affect TDP-43 expression in astrocytes, leading to reduced TDP-43 level that appears to underly progressive neurodegeneration in cortex and spinal cord, motor dysfunction, paralysis and premature death without the appearance of TDP-43 inclusions (Yang et al. 2014).

A key aspect not modelled in conditional KO mice, or any other current mouse model displaying TDP-43 loss of function, such as the rNLS mice, is the contribution of human-specific CE such as those found within *STMN2, UNC13A* or *ATG4B*, that appear to play critical roles in disease pathogenesis (Mehta et al. 2023). As CE are not well conserved between mouse and humans, to study the contributions of human-specific CE, it is necessary to develop humanised models, such as the already established in *STMN2* model (Baughn et al. 2023), carrying human-specific CE and to bred them onto a loss of TDP-43 function context– as in conditional KO models, or achieved with treatments such as antisense oligonucleotides (ASOs) against *Tardbp*. Future studies with different humanised CE models will be required to dissect their contribution towards disease pathogenesis, and they will also be extremely useful for the in vivo testing of therapeutics such as ASOs aiming at silencing specific human CE expression.

### *TARDBP* genomically humanised models

We recently developed a genomically humanised *TARDBP* model, in which the endogenous mouse *Tardbp* gene has been replaced by the human *TARDBP* orthologue within its correct locus from the start ATG to the stop codon, including all human introns in between (Devoy et al. 2021). This is the first model expressing only human TDP-43, without expression from endogenous mouse alleles, as, to our knowledge, all attempts to rescue the lethality of *Tardbp* KO with transgenic models have been unsuccessful. These genomically humanised mice are useful to assess the pathogenicity of human mutations when expressed at endogenous levels, as well as uniquely suited for the in vivo testing of therapeutic strategies aimed at interfering with the human *TARDBP* gene, such as ASOs. Moreover, they should be helpful to study possible human-specific characteristics of the human TDP-43 protein, including the possibility of human-specific TDP-43 pathogenic folds.

## Models developing TDP-43 pathology by not primarily targeting *Tardbp*/TDP-43

An important point to consider is that as *TARDBP* mutations are rare (around 4% of familial cases and up to 1% of sporadic ALS) (Renton et al. 2014), in the great majority of patients, TDP-43 pathology is defined by the exclusion from the nucleus and the cytoplasmic and/or nuclear aggregation of WT TDP-43. Thus, a complementary strategy to model TDP-43 pathology is to remove WT TDP-43 from the nucleus by different approaches, including different stress treatments or by mutating genes/pathways involved in TDP-43 proteinopathies other than TDP-43 itself. Here, we provide a few examples of published treatments and gene mutations or combinations thereof that lead to TDP-43 pathology (Table 2).

**Table 2:**
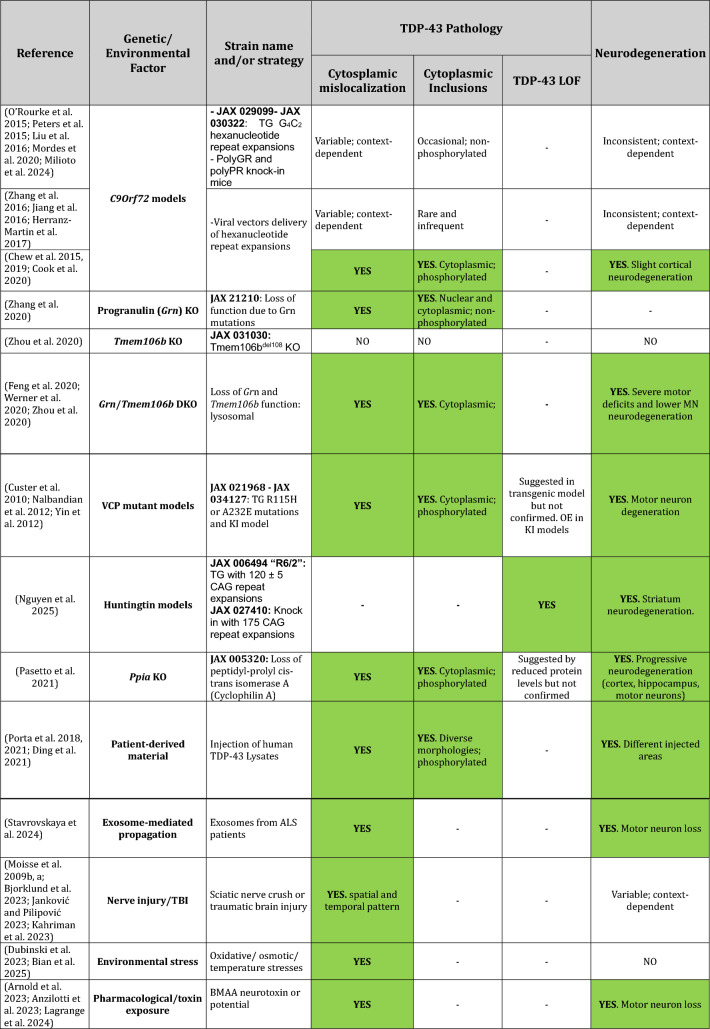
Models developing TDP-43 pathology by not primarily targeting *Tardbp*/TDP-43

Models are categorized based on the genetic or environmental factor involved, and the corresponding TDP-43 mislocalization, inclusion characteristics (including phosphorylation status), and presence of neurodegeneration are detailed.

TG, transgenic; C9Orf72, chromosome 9 open reading frame 72, BAC, bacterial artificial chromosome; GRN, progranulin; KI, knock in; KO, knockout; DKO, double knockout; VCP, valosin-containing protein, PPIA, cyclophilin A/peptidyl-prolyl cis–trans isomerase A; ALS, amyotrophic lateral sclerosis; TBI, traumatic brain injury; BMAA, β-N-methylamino-L-alanine; –, not assessed; *OE*, over expression

### Models developing TDP-43 pathology by targeting other genes

Hexanucleotide repeat expansions in the chromosome 9 open reading frame 72 gene (*C9Orf72*) are the most common genetic cause of FTD and ALS. Transgenic, viral expression or KI models of G4C2 repeat expansions in rodents are widely used for studying FTD and ALS. Most of these transgenic and KI models have not shed much light on the relationship between repeat expansions and TDP-43 pathology, with a majority of transgenic and KI models showing no TDP-43 pathology (O’Rourke et al. 2015; Peters et al. 2015; Milioto et al. 2024), although a few have TDP-43 pathology and inclusions (Liu et al. 2016). Of the viral models, some develop occasional TDP-43 pathology (Zhang et al. 2016; Jiang et al. 2016; Herranz-Martin et al. 2017), while others, particularly polyGR models, present with extensive TDP-43 pathology, leading to colocalization of TDP-43 with polyGR aggregates (Chew et al. 2015, 2019; Cook et al. 2020).

Mutations in progranulin (*GRN*) are a major cause of FTLD with TDP-43 pathology (Irwin et al. 2015). As mutations lead to loss of function, *Grn* KO mice have been used to study disease pathogenesis. Indeed, *Grn* deficiency leads to TDP-43 cytoplasmic mislocalization and the accumulation of nuclear and cytoplasmic TDP-43 inclusions (Zhang et al. 2020). Conversely, *TMEM106B,* a locus associated with modifying FTLD-TDP risk (Van Deerlin et al. 2010), encodes TMEM106B fibrils that have been found as a major component of inclusions in FTLD-TDP cases; these fibrils are present in different FTLD-TDP patients as well as in a wide range of other neurodegenerative disorders, including LATE, synucleopathies, or tauopathies amongst others (Perneel et al. 2023), together with the ageing brain (Schweighauser et al. 2022). *TMEM106B* is an established risk factor for FTLD caused by *GRN* mutations, with both proteins being required for correct lysosome function. Interestingly, double KO mice (*Grn*^*−/−*^; *Tmem106b*^*−/−*^*)* clearly exacerbate disease phenotypes present in each individual KO, leading to enhanced lysosome abnormalities underlying severe motor deficits and neurodegeneration (Feng et al. 2020; Werner et al. 2020; Zhou et al. 2020). Remarkably, these double KO develop *bona fide* TDP-43 inclusions of yet unknown folds, but without accompanied TDP-43 nuclear clearance, making them excellent models to study the consequences of TDP-43 cytoplasmic deposition, and the possible study of mouse TDP-43 folds in the context of lysosomal dysfunction.

Another prominent example are mice carrying VCP mutations. *VCP* mutations can produce heterogeneous clinical presentations with TDP-43 pathology, including ALS, FTLD, inclusion body myopathy with Paget's disease of the bone and multisystem proteinopathy. VCP is a multifunctional protein essential for cellular homeostasis, with prominent roles in multiple pathways including autophagy and the degradation of ubiquitinated proteins to ensure correct proteostasis (Chu et al. 2023). Several models are available, including transgenic models expressing human mutations and also KI mice. In particular, transgenic models expressing human R115H or A232E mutations lead to TDP-43 cytoplasmic accumulation in the form of inclusions, motor neuron degeneration and TDP-43 nuclear exit, although it is not yet clear if this is enough to trigger the expression of CE (Custer et al. 2010). A KI model carrying the R115H mutation in heterozygosis also develops TDP-43 pathology and motor neuron loss, with a concomitant increase in TDP-43 protein levels that, although not tested, might lead to the expression of skiptic exons (Yin et al. 2012), whereas the R115H mutation in homozygosis leads to accelerated VCP associated disease and increased TDP-43 pathology (Nalbandian et al. 2012). Overall, these models are useful for studying the effects of TDP-43 pathology in the context of generalized proteostasis abnormalities.

Interestingly, other neurodegenerative disorders present with secondary TDP-43 pathology. In the R6/2 and Q175 mouse models of Huntington's disease (HD), TDP-43 pathology leads to loss of splicing function. In R6/2 mice, this is evidenced by aberrant splicing patterns that overlap with those seen after TDP-43 knockdown. Similarly, Q175 KI mice show progressive nuclear TDP-43 loss, accompanied by splicing changes (Nguyen et al. 2025). These findings provide further evidence that TDP-43 dysfunction contributes to the mis-processing of mRNA in a variety of neurodegenerative disorders.

An additional recent example is the Cyclophilin A/peptidyl-prolyl cis–trans isomerase A (PPIA) KO mouse. Despite its abundance in the central nervous system, the primary function of PPIA remains largely undefined, although it is known to act as a molecular chaperone and interacts with TDP-43 and other heterogenous nuclear ribonucleoproteins, with PPIA levels being reduced in peripheral blood from ALS patients (Luotti et al. 2020). Constitutive *Ppia* KO mice have an increase in phosphorylated cytoplasmic TDP-43 inclusions, accompanied by a significant reduction in its protein level, although if this is enough to lead to CE expression has not yet been assessed (Pasetto et al. 2021). These pathological changes are associated with progressive neurodegeneration primarily affecting cortex and hippocampus and later motor neurons, suggesting that PPIA plays a critical role in maintaining TDP-43 homeostasis and neuronal integrity, whilst providing an excellent model to study the consequences of TDP-43 pathology.

### Patient post-mortem material models

A different strategy to develop TDP-43 pathology in mice is the direct injection of material obtained from patients into different brain regions using stereotaxic equipment. Indeed, extracts obtained from patient post-mortem material have been used to develop models with TDP-43 pathology, particularly to study propagation at different timepoints post-injection. Studies using transgenic mice expressing hTDP-43, such as the rNLS8 model, show that lysates from FTD-TDP patients can be transmitted in a progressive manner through the brain connectome when injected into different areas. Interestingly, human extracts are also able to propagate mouse TDP-43 pathology in WT mice, although to a lesser extent than hTDP-43 (Porta et al. 2018). Moreover, different TDP-43 strains from FTLD-TDP subtypes can induce morphologically diverse TDP-43 aggregates and spreading patterns (Porta et al. 2021). Furthermore, in vitro preformed TDP-43 fibrils injected into the primary motor cortex have been shown to lead to TDP-43 propagation in mice expressing human WT TDP-43, followed by the appearance of motor deficits including electromyogram abnormalities, supporting the anterograde transmission of TDP-43 pathology from the cortex to the spinal cord, affecting the entire motor unit (Ding et al. 2021). Although in these models TDP-43 cytoplasmic inclusions are present and can be transmissible, no data was presented to assess possible effects on TDP-43 nuclear function, such as in splicing. These strategies are likely to be used in attempts to develop models with different TDP-43 folds.

In addition, exosome-mediated propagation has emerged as another mechanism that might contribute to TDP-43 pathology. Mice receiving exosomes from cerebrospinal fluid obtained from ALS patients developed a significant loss of motor neurons in the spinal cord accompanied by increased microglial activation and TDP-43 mislocalisation (Stavrovskaya et al. 2024). These findings highlight the potential role of exosome-mediated propagation of TDP-43 pathology.

### Treatment models

Apart from targeting other genes involved in TDP-43 proteinopathies and treating with extracts from patient post-mortem material, there are other paradigms known to lead to TDP-43 nuclear exit by affecting neuronal functions, including axotomies or traumatic brain injury. After nerve damage, such as crushing of the sciatic nerve, TDP-43 exits the motor neuron nucleus and accumulates at the axonal site of injury, increasing its expression levels until coming back into the nucleus after damage recovery (Moisse et al. 2009b, a). In traumatic brain injury (TBI), a similar pattern can be observed, with TDP-43 exiting the nucleus and accumulating at the site of injury in acute concussion as well as in mild traumatic models (Janković and Pilipović 2023). Furthermore, this mislocalisation follows a spatial and temporal pattern (Bjorklund et al. 2023). Interestingly, in a *C9Orf72* mouse model, mild repeated trauma has been shown to lead, months after the treatment, to extensive neuronal loss and TDP-43 pathology, including cytoplasmic aggregation and TDP-43 nuclear exit, although it is not yet clear if CE were expressed, overall suggesting a role for brain injury in the development of TDP-43 pathology (Kahriman et al. 2023).

This behaviour is reminiscent of TDP-43 exiting the nucleus in response to different kinds of stresses in cellular models, such as oxidative, osmotic or temperature stresses. Although TDP-43, as other RNA binding proteins, can exit the nucleus under different stress conditions in vitro and in vivo, accumulating in the cytoplasm including colocalizing with stress granules, it is not yet understood what the functional consequences of this nuclear exit and site of damage accumulation are. It is possible that failure to clear TDP-43 from the cytoplasm might lead to pathological changes in TDP-43 aggregation propensity leading to solid phase transitions that might act as precursors of inclusions (Gasset-Rosa et al. 2019). Attempts to recapitulate TDP-43 nuclear exit from the nucleus by different stress conditions in vivo in the mouse include focal cerebral ischemia stroke, or high temperature shock. Using a transient middle cerebral artery occlusion model, an increase in TDP-43 positive staining with cytoplasmic accumulation was observed, highlighting a potential link between ischaemic stroke and TDP-43 proteinopathy (Bian et al. 2025). Hyperthermia can also induce mislocalisation of TDP-43 from the nucleus to the cytoplasm, particularly in mice with TDP-43 mutations (Dubinski et al. 2023). However, it is not yet clear what are the long-term consequences of these treatments in vivo, and if they might affect TDP-43 nuclear functions.

In terms of pharmacological or toxin treatments, an example comes from the exposure to β-N-methylamino-L-alanine (BMAA), a neurotoxin present in some cyanobacteria that has been linked to the development of an ALS, Parkinsonism and dementia complex also named as Guam-ALS. Injection of BMAA can lead to motor deficits when in WT mice (Anzilotti et al. 2023) and a TDP-43 transgenic model (Arnold et al. 2023). Recently, a study suggested a link between the high local incidence of ALS cases in a French Alps village to the consumption of a toxin (gyromitrin) found in consumed fungi (Lagrange et al. 2024). Even though gyromitrin has not been tested in animal models in relation to ALS yet, these and other possible future findings that point to environmental risk factors for ALS must be carefully examined in animals in connection to the onset of TDP-43 pathology.

Overall, the development of different in vivo treatments promoting TDP-43 nuclear exit and cytoplasmic aggregation represents an interesting area for future modelling of sporadic ALS, that could be aided by the use of predisposed models, such as presymptomatic TDP-43 mutant mice, for developing multiple hits models.

## Future perspectives and conclusions

There is clearly an urgent need to develop more models recapitulating as closely as possible the complexities of the human TDP-43 proteinopathies. Ideally, these will include mouse models but will also require working with a combination of other animals (worms, flies, fish etc.) and a variety of cellular models such as induced pluripotent stem cells (iPSCs) derived from patients or carrying known pathogenic mutations, organoids or 2D/3D models amongst others, with ultimate validation in patient-derived material.

Outstanding issues in the field that will be aided by such mouse models include, the possibility of testing causality for the different TDP-43 folds, the roles of specific CE in the disease cascade, the dissection of the contributions of the two main aspects of TDP-43 pathology (nuclear exit and cytoplasmic accumulation), and the understanding of the differential vulnerabilities of specific neuronal (and non-neuronal) populations in the different TDP-43 proteinopathies. To start to address these issues, new mouse models are required together with thoughtful breeding of combinations of existing strains. Moreover, as the presence of TDP-43 pathology is the main unifying feature of ALS, the development of much needed mouse models of sporadic ALS and other TDP-43 proteinopathies will entail creating combinations of treatments and/or gene modifications starting a cascade of events eventually leading to TDP-43 nuclear exit and its cytoplasmic aggregation, ideally in a subset of animals. A plausible strategy to achieve this might be to combine different gene modifications in TDP-43 itself, leading to its presymptomatic dysregulation, with other treatments aiming at disrupting different key pathways in cellular populations such as motor neurons, glia or skeletal muscle that are thought to be critical in the stepwise nature of ALS pathogenesis processes, following the lessons from multiple hit cancer models (Larrayoz et al. 2023).

Overall, the difficulties in generating mouse strains modelling as closely as possible the human TDP-43 proteinopathies probably reflect the fact that these pathologies are very complex and heterogenous in patients. However, new forthcoming models, together with the existing ones reviewed here, will be critical to further our understanding of disease processes.

## Data Availability

No datasets were generated or analysed during the current study.
